# Life history strategies among soil bacteria—dichotomy for few, continuum for many

**DOI:** 10.1038/s41396-022-01354-0

**Published:** 2023-02-02

**Authors:** Bram W. G. Stone, Paul Dijkstra, Brianna K. Finley, Raina Fitzpatrick, Megan M. Foley, Michaela Hayer, Kirsten S. Hofmockel, Benjamin J. Koch, Junhui Li, Xiao Jun A. Liu, Ayla Martinez, Rebecca L. Mau, Jane Marks, Victoria Monsaint-Queeney, Ember M. Morrissey, Jeffrey Propster, Jennifer Pett-Ridge, Alicia M. Purcell, Egbert Schwartz, Bruce A. Hungate

**Affiliations:** 1 Earth and Biological Sciences Directorate, Pacific Northwest National Lab, Richland, WA USA; 2 Center for Ecosystem Science and Society, Northern Arizona University, Flagstaff, AZ USA; 3 Department of Biological Sciences, Northern Arizona University, Flagstaff, AZ USA; 4 Department of Ecology and Evolutionary Biology, University of California, Irvine, CA USA; 5 Department of Agronomy, Iowa State University, Ames, IA USA; 6 APC Microbiome Ireland and School of Microbiology, University College Cork, Cork, Ireland; 7 Institute for Environmental Genomics, Department of Microbiology and Plant Biology, University of Oklahoma, Norman, OK USA; 8 Division of Plant and Soil Sciences, West Virginia University, Morgantown, WV USA; 9 Physical and Life Sciences Directorate, Lawrence Livermore National Lab, Livermore, CA USA; 10 Life and Environmental Sciences Department, University of California Merced, Merced, CA USA; 11 Department of Biological Sciences, Texas Tech University, Lubbock, TX USA

**Keywords:** Microbial ecology, Stable isotope analysis, Soil microbiology

## Abstract

Study of life history strategies may help predict the performance of microorganisms in nature by organizing the complexity of microbial communities into groups of organisms with similar strategies. Here, we tested the extent that one common application of life history theory, the copiotroph-oligotroph framework, could predict the relative population growth rate of bacterial taxa in soils from four different ecosystems. We measured the change of in situ relative growth rate to added glucose and ammonium using both ^18^O–H_2_O and ^13^C quantitative stable isotope probing to test whether bacterial taxa sorted into copiotrophic and oligotrophic groups. We saw considerable overlap in nutrient responses across most bacteria regardless of phyla, with many taxa growing slowly and few taxa that grew quickly. To define plausible life history boundaries based on in situ relative growth rates, we applied Gaussian mixture models to organisms’ joint ^18^O–^13^C signatures and found that across experimental replicates, few taxa could consistently be assigned as copiotrophs, despite their potential for fast growth. When life history classifications were assigned based on average relative growth rate at varying taxonomic levels, finer resolutions (e.g., genus level) were significantly more effective in capturing changes in nutrient response than broad taxonomic resolution (e.g., phylum level). Our results demonstrate the difficulty in generalizing bacterial life history strategies to broad lineages, and even to single organisms across a range of soils and experimental conditions. We conclude that there is a continued need for the direct measurement of microbial communities in soil to advance ecologically realistic frameworks.

## Introduction

The concept of copiotrophy and oligotrophy in microbial communities offers the potential for an organizing principle to describe the complexity of microbial systems. As such, it has been discussed for decades in relation to marine environments [1] and soils [2]. The framework is a direct descendent of r-K selection theory, which has long been applied to larger organisms [3]. If successful, life history strategy frameworks should support inferences about processes from patterns in taxonomic composition. The copiotroph-oligotroph framework posits that microorganisms, facing strong selective pressure from their environment, adapt strategies defined by two endpoints along a continuum: either growing and reproducing quickly in the presence of abundant nutrients (copiotrophs), or specializing in resource-poor niches to escape from competition (oligotrophs) [4, 5]. Evidence for this framework in soils was first presented from greater relative abundance of some bacterial phyla in response to sucrose addition, indicating copiotrophic strategies, whereas other bacterial phyla were either unresponsive or responded negatively, suggesting oligotrophic strategies [6]. An expansion of growth-trait associated strategies centers around the trade-off between maintenance energy, growth efficiency (i.e., yield), and growth rate [7, 8] or in the investment in resource acquisition [9, 10].

**Fig. 1:**
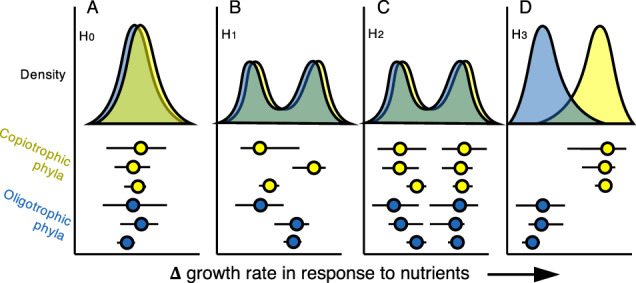
Hypothesized patterns of nutrient response across microbial lineages and associated life history strategy distributions. Points at the bottom represent a measure of centrality of nutrient responses across taxa within a phylum while lines represent a measure of spread. Phylum-specific responses are summarized by an expected density distribution of each life history strategy above. Note that the heights of density distributions for each strategy were made equal only for visibility. Panels show nutrient response under different hypotheses. **A** H_0_ a continuum of responses exists across all phyla. **B** H_1_ bimodality across the community will occur regardless of life history strategy due to differences across phyla (i.e., phyla-level classifications are inaccurate). **C** H_2_ bimodality across the community will occur regardless of life history strategy due to differences across taxa within each phylum (i.e., phyla-level classifications are imprecise). **D** H_3_ that phyla show clear tendencies for one strategy or another.

The copiotroph-oligotroph framework is commonly used for the interpretation of 16S rRNA gene bacterial community data [11–13]. Fierer et al. explicitly emphasized continuous and taxon-specific behavior, “These results do not suggest that every member of the Acidobacteria, β-Proteobacteria, and Bacteroidetes phyla are distinctly copiotrophic or oligotrophic” [6]. Thus, the proposed framework in microbial ecology describes a continuum of nutrient responses. Viewed as a continuum, the copiotroph-oligotroph hypothesis holds that the traits associated with these two life history strategies are negatively correlated—not mutually exclusive—and that a continuous range of responses is expected. Additionally, factors beyond the physiological potential of the microorganism or available resources can influence population growth. Factors such as viral predation [14] or soil structure [15, 16] are relevant to understanding microbal behavior in the soil habitat but may lead to a higher frequency of intermediate responses to nutrient addition (Fig. 1A). Such an outcome would raise serious objections to the usefulness of a dichotomous categorization scheme as a tool to understand and predict microbial communities in soil.

It is also possible that the pressure to select for either copiotrophic or oligotrophic strategies is strong in soils but that the current practice of assignment of bacterial phyla to one strategy or the other is inaccurate (Fig. 1B) or imprecise (Fig. 1C). For example, classifying individual phyla as oligotrophic or copiotrophic may not be correct or useful given the behavior of the majority of their constituent taxa (i.e., categorization is inaccurate). Further, taxa within a given phylum may show a mix of response types (i.e., categorization is imprecise) according to previous publications [17, 18]. For example, if we assume that microorganisms must invest in one or the other growth strategy exclusively, we would expect to observe a bimodal distribution of growth responses in copiotrophic and a bimodal distribution in oligotrophic taxa. Although microbial ecologists typically organize their conclusions at the phylum level – it is well understood that bacterial lineages within phyla can have distinct metabolic and ecological roles and that finer taxonomic resolution may be necessary for assigning strategies accurately [17, 19–23]. This would indicate that current categorizations should be updated based on new methods for measuring taxon-specific growth rate or nutrient response, or that finer levels of taxonomic organization are more appropriate for making life history assignments as recommended by Ho and colleagues (based on references therein) [23]. By contrast, if the life history strategy is coherent at the phylum level, and current classifications are correct, we expect to see a clear distinction in the distributions of growth response between copiotrophic and oligotrophic phyla (Fig. 1D).

Here, we analyzed published quantitative stable isotope probing (qSIP) data [21, 24, 25] on the growth of bacterial taxa within the complex and heterogenous soil environment. Because microbial activity is strongly dependent on resource stoichiometry [26–28], we analyzed data from two experimental treatments – labile carbon (C) or carbon + ammonium (N) addition. This design was meant to minimize N limitation in at least one treatment. We applied phylum-level assignments of life history strategy from the microbiological literature (Table 1) and compared the expected responses of individual bacterial taxa to their growth responses measured by isotopic enrichment. Given previous findings of taxon-specific responses [17, 18], we compared nutrient responses at different levels of taxonomic organization from the level of phylum to genus. Our hypotheses were (see Fig. 1):

**Table 1:** Classification of microbial life history strategies of major microbial lineages.

Representative phylum or subphylum	Times cited as copiotroph	Times cited as oligotroph	Expected life history
Alphaproteobacteria	3	4	Oligotroph
Betaproteobacteria	4	0	Copiotroph
Gammaproteobacteria	6	1	Copiotroph
Deltaproteobacteria	0	1	Oligotroph
Acidobacteria	0	8	Oligotroph
Firmicutes	3	1	Copiotroph
Bacteroidetes	5	2	Copiotroph
Actinobacteria	4	2	Copiotroph
Verrucomicrobia	0	5	Oligotroph
Planctomycetes	0	5	Oligotroph
Chloroflexi	0	1	Oligotroph
Gemmatimonadetes	1	0	Copiotroph
Cyanobacteria	0	1	Oligotroph

Classifications of expected behavior are based off the frequency that lineages within a given representative phylum (or subphylum) was cited as following copiotroph-associated behavior or oligotroph-associated behavior as summarized by Ho et al. [23]. 10.1093/femsec/fix006.

H_0_, Null: No bimodality in nutrient response of taxa to nutrient addition (i.e., no clear selection for one strategy or the other exists, Fig. 1A)

H_1_, misclassification: Bimodality in nutrient responses to nutrient addition within each life history category (i.e., phyla show inherent tendencies for one strategy or the other, but have been misclassified, Fig. 1B)

H_2_, taxonomic resolution: Bimodality in nutrient responses to nutrient addition within each life history category (i.e., groups of taxa show inherent tendencies for one strategy or the other, but only at a finer taxonomic resolution than the phylum level, Fig. 1C)

H_3_, phylum-level traits: Significant differences in nutrient response to nutrient addition between life history strategies (i.e., clear tendencies for one strategy or another; current phylum-level assignments of life history strategies are supported, Fig. 1D)

## Materials and methods

Data were analyzed from samples collected, processed, and published previously [21, 25, 29] and have been summarized here. The present analysis, which consisted of sequence data processing, the calculation of taxon-specific isotopic signatures, and subsequent analyses, reflects original work.

### Sample collection and isotope incubation

To generate experimental data, three replicate soil samples were collected from the top 10 cm of plant-free patches in four ecosystems along the C. Hart Merriam elevation gradient in Northern Arizona. From low to high elevation, these sites are located in the following environments: desert grassland (GL; 1760 m), piñon-pine juniper woodland (PJ; 2020 m), ponderosa pine forest (PP; 2344 m), and mixed conifer forest (MC; 2620 m). Soil samples were air-dried for 24 h at room temperature, homogenized, and passed through a 2 mm sieve before being stored at 4 °C for another 24 h. This produced three distinct but homogenous soil samples from each of the four ecosystems that were subject to experimental treatments. Three treatments were applied to bring soils to 70% water-holding capacity: water alone (control), water with glucose (C treatment; 1000 µg C g^−1^ dry soil), or water with glucose and a nitrogen source (CN treatment; [NH_4_]_2_SO_4_ at 100 µg N g^−1^ dry soil). To track growth through isotope assimilation, both ^18^O-enriched water (97 atom %) and ^13^C-enriched glucose (99 atom %) were used. In all treatments isotopically heavy samples were paired with matching “light” samples that received water with a natural abundance isotope signatures. For ^18^O incubations, this design resulted in three soil samples per ecosystem per treatment (across four ecosystems and three treatments, *n* = 36) while ^13^C incubations were limited to only C and CN treatments (*n* = 24). Previous analyses suggest that three replicates is sufficient to detect growth of 10 atom % ^18^O in microbial DNA with a power of 0.6 and a growth of 5 atom % ^18^O with a power of 0.3 (12 and 6 atom % respectively for ^13^C) [30]. All soils were incubated in the dark for one week. Following incubation, soils were frozen at −80 °C for one week prior to DNA extraction.

### Quantitative stable isotope probing

The procedure of qSIP (quantitative stable isotope probing) is described here but has been applied to these samples as previously published [17, 21, 25]. DNA extraction was performed on soils using a DNeasy PowerSoil HTP 96 Kit (MoBio Laboratories, Carlsbad, CA, USA) and following manufacturer’s protocol. Briefly, 0.25 g of soils from each sample were carefully added to deep, 96-well plates containing zirconium dioxide beads and a cell lysis solution with sodium dodecyl sulfate (SDS) and shaken for 20 min. Following cell lysis, supernatant was collected and centrifuged three times in fresh 96-well plates with reagents separating DNA from non-DNA organic and inorganic materials. Lastly, DNA samples were collected on silica filter plates, rinsed with ethanol and eluted into 100 µL of a 10 mM Tris buffer in clean 96-well plates. To quantify the degree of ^18^O or ^13^C isotope incorporation into bacterial DNA (excess atom fraction or EAF), the qSIP protocol [31] was used, though modified slightly as reported previously [21, 24, 32]. Briefly, microbial growth was quantified as the change in DNA buoyant density due to incorporation of the ^18^O or ^13^C isotopes through the method of density fractionation by adding 1 µg of DNA to 2.6 mL of saturated CsCl solution in combination with a gradient buffer (200 mM Tris, 200 mM KCL, 2 mM EDTA) in a 3.3 mL OptiSeal ultracentrifuge tube (Beckman Coulter, Fullerton, CA, USA). The solution was centrifuged to produce a gradient of increasingly labeled (heavier) DNA in an Optima Max bench top ultracentrifuge (Beckman Coulter, Brea, CA, USA) with a Beckman TLN-100 rotor (127,000 × *g* for 72 h) at 18 °C. Each post-incubation sample was thus converted from a continuous gradient into approximately 20 fractions (150 µL) using a modified fraction recovery system (Beckman Coulter). The density of each fraction was measured with a Reichart AR200 digital refractometer (Reichert Analytical Instruments, Depew, NY, USA). Fractions with densities between 1.640 and 1.735 g cm^−3^ were retained as densities outside this range generally did not contain DNA. In all retained fractions, DNA was cleaned and purified using isopropanol precipitation and the abundance of bacterial 16S rRNA gene copies was quantified with qPCR using primers specific to bacterial 16S rRNA genes (*Eub* 515F: AAT GAT ACG GCG ACC ACC GAG TGC CAG CMG CCG CGG TAA, 806R: CAA GCA GAA GAC GGC ATA CGA GGA CTA CVS GGG TAT CTA AT). Triplicate reactions were 8 µL consisting of 0.2 mM of each primer, 0.01 U µL^−1^ Phusion HotStart II Polymerase (Thermo Fisher Scientific, Waltham, MA), 1× Phusion HF buffer (Thermo Fisher Scientific), 3.0 mM MgCl_2_, 6% glycerol, and 200 µL of dNTPs. Reactions were performed on a CFX384 Touch Real-Time PCR Detection System (Bio-Rad, Hercules, CA, USA) under the following cycling conditions: 95 °C at 1 min and 44 cycles at 95 °C (30 s), 64.5 °C (30 s), and 72 °C (1 min). Separate from qPCR, retained sample-fractions were subject to a similar amplification step of the 16S rRNA gene V4 region (515F: GTG YCA GCM GCC GCG GTA A, 806R: GGA CTA CNV GGG TWT CTA AT) in preparation for sequencing with the same reaction mix but differing cycle conditions – 95 °C for 2 min followed by 15 cycles at 95 °C (30 s), 55 °C (30 s), and 60 °C (4 min). The resulting 16S rRNA gene V4 amplicons were sequenced on a MiSeq sequencing platform (Illumina, Inc., San Diego, CA, USA). DNA sequence data and sample metadata have been deposited in the NCBI Sequence Read Archive under the project ID PRJNA521534.

### Sequence processing and qSIP analysis

Independently from previous publications, we processed raw sequence data of forward and reverse reads (FASTQ) within the QIIME2 environment [33] (release 2018.6) and denoised sequences within QIIME2 using the DADA2 pipeline [34]. We clustered the remaining sequences into amplicon sequence variants (ASVs, at 100% sequence identity) against the SILVA 138 database [35] using a pre-trained open-reference Naïve Bayes feature classifier [36]. We removed samples with less than 3000 sequence reads, non-bacterial lineages, and global singletons and doubletons. We converted ASV sequencing abundances in each fraction to the number of 16S rRNA gene copies per gram dry soil based on qPCR abundances and the known amount of dry soil equivalent added to the initial extraction. This allowed us to express absolute population densities, rather than relative abundances. Across all replicates, we identified 114 543 unique bacterial ASVs.

We calculated the ^18^O and ^13^C excess atom fraction (EAF) for each bacterial ASV using R version 4.0.3 [37] and data.table [38] with custom scripts available at https://www.github.com/bramstone/. Negative enrichment values were corrected using previously published methods [17]. ASVs that appeared in less than two of the three replicates of an ecosystem-treatment combination (*n* = 3) and less than three density fractions within those two replicates were removed to avoid assigning spurious estimates of isotope enrichment to infrequent taxa. Any ASVs filtered out of one ecosystem-treatment group were allowed to be present in another if they met the frequency threshold. Applying these filtering criteria, we limited our analysis towards 3759 unique bacterial ASVs which accounted for a small proportion of the total diversity but represented 68.0% of all sequence reads, and encompassed most major bacterial groups (Supplementary Fig. 1).

### Analysis of life history strategies and nutrient response

All statistical tests were conducted in R version 4.0.3 [37]. We assessed the ability of phylum-level assignment of life history strategy to predict growth in response to C and N addition, as proxied by the incorporation of heavy isotope during DNA replication [39, 40]. Phylum-level assignments (Table 1) were based on the most frequently observed behavior of lineages with a representative phylum (or subphylum) as compiled previously [23]. We averaged ^18^O EAF values of bacterial taxa for each treatment and ecosystem and then subtracted the values in control soils from values in C-amended soils to determine C response (∆^18^O EAF_C_) and from the ^18^O EAF of bacteria in CN-amended soils to determine C and N response (Δ^18^O EAF_CN_). Because an ASV must have a measurable EAF in both the control and treatment for a valid Δ^18^O EAF to be calculated, we were only able to resolve the nutrient response for 2044 bacterial ASVs – 1906 in response to C addition and 1427 in response to CN addition.

We used Gaussian finite mixture modeling, as implemented by the mclust R package [41], to demarcate plausible multi-isotopic signatures for oligotrophs and copiotrophs. For each treatment, we calculated average per-taxon ^13^C and ^18^O EAF values. To compare both isotopes directly, we divided ^18^O EAF values by 0.6 based on the estimate that this value (designated as *µ*) represents the fraction of oxygen atoms in DNA derived from the ^18^O-water, rather than from ^16^O within available C sources [42]. Two mixture components, corresponding to oligotrophic and copiotrophic growth modes, were defined using the Mclust function using ellipsoids of equal volume and shape. We observed several microorganisms with high ^18^O enrichment but comparatively low ^13^C enrichment, potentially indicating growth following the depletion of the added glucose, and that were reasonably clustered as oligotrophs in our mixture model.

We tested how frequently mixture model clustering of each microorganism’s growth (based on average ^18^O–^13^C EAF in a treatment) could predict its growth across replicates (*n* = 12 in each treatment—although individual). We applied the treatment-level mixture models defined above to the per-taxon isotope values in each replicate, recording when a microorganism’s life history strategy in a replicate agreed with the treatment-level cluster, and when it didn’t. We used exact binomial tests to test whether the number of “successes” (defined as a microorganism being grouped in the same life history category as its treatment-level cluster) was statistically significant. To account for type I error across all individual tests (one per ASV per treatment), we adjusted *P* values in each treatment using the false-discovery rate (FDR) method [43].

To determine the extent that life history categorizations may be appropriately applied at finer levels of taxonomic resolution, we constructed several hierarchical linear models using the lmer function in the nlme package version 3.1-149 [44]. To condense growth information from both isotopes into a single analysis, ^18^O and ^13^C EAF values were combined into a single variable using principal components analysis separately for each treatment. Across the C and CN treatments, the first principal component (PC1) was able to explain – respectively – 86% and 91% of joint variation of ^18^O and ^13^C EAF values. In all cases, we applied PC1 as the response variable and treated taxonomy and ecosystem as random model terms to limit the potential of pseudo-replication to bias significance values. We used likelihood ratio analysis and Akaike information criterion (AIC) values to compare models where life history strategy was determined based on observed nutrient responses at different taxonomic levels (Eq. 1) against a model with the same random terms but without any life history strategy data (Eq. 2). Separate models were applied to each treatment. To reduce model overfitting, we removed families represented by fewer than three bacterial ASVs as well as phyla represented by only one order. In addition, we removed bacterial ASVs with unknown taxonomic assignments (following Morrissey et al. [21]). This limited our analysis to 1 049 ASVs in the C amendment and 984 in the CN amendment.

1 $${{{{{\rm{PC}}}}}}{1}_{{18{{{{{\rm{O}}}}}} - 13{{{{{\rm{C}}}}}}}}\sim {{{{{\rm{strategy}}}}}} + 1|{{{{{\rm{phylum}}}}}}/{{{{{\rm{class}}}}}}/{{{{{\rm{order}}}}}}/{{{{{\rm{family}}}}}}/{{{{{\rm{genus}}}}}}/{{{{{\rm{eco}}}}}}$$

2 $${{{{{\rm{PC}}}}}}{1}_{{18{{{{{\rm{O}}}}}} - 13{{{{{\rm{C}}}}}}}}\sim 1 + 1|{{{{{\rm{phylum}}}}}}/{{{{{\rm{class}}}}}}/{{{{{\rm{order}}}}}}/{{{{{\rm{family}}}}}}/{{{{{\rm{genus}}}}}}/{{{{{\rm{eco}}}}}}$$

Here, life history strategy was defined at each taxonomic level using the mixture models above and based on the mean ^18^O and ^13^C EAF values of each bacterial lineage (Supplemental Fig. 2). We compared these models with the no-strategy model (Eq. 2) directly using likelihood ratio testing.

## Results

### Bacteria with strongly positive short-term nutrient response represent a small proportion of diversity within a limited number of phyla

**Fig. 2:**
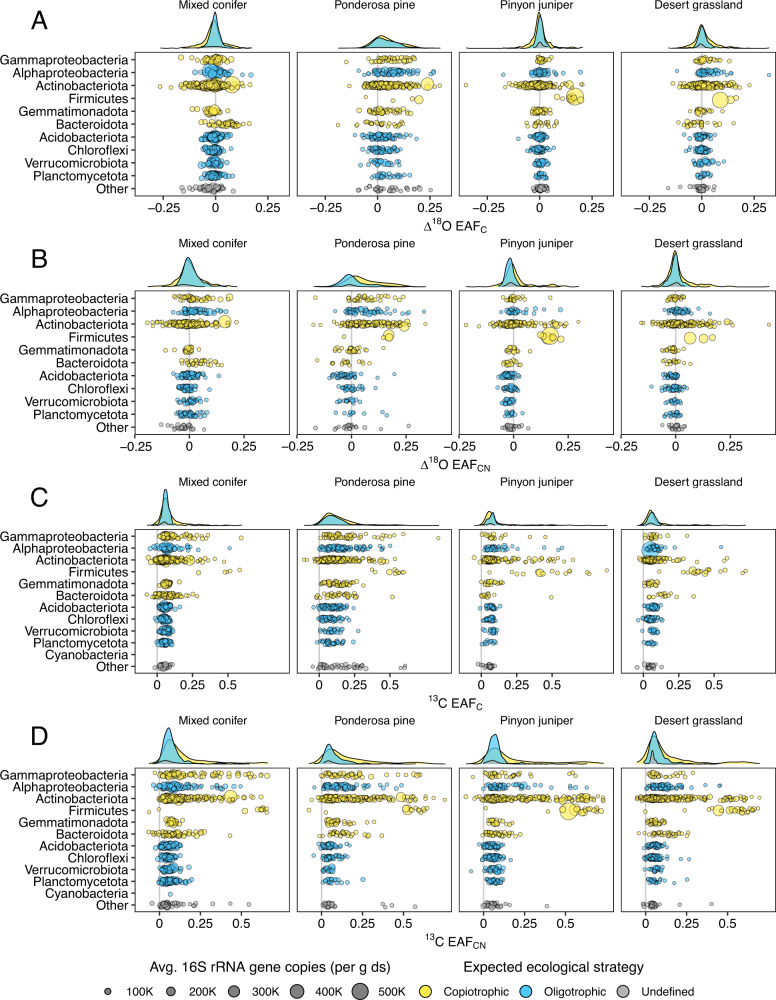
Bacterial response to nutrient addition across phyla and life history groups. Each point represents the mean isotopic enrichment (excess atom fraction or EAF) for an individual bacterial taxon across three soil replicates, organized by bacterial phylum. Phyla are colored by the categorical assignment of life history strategy applied at the phylum level taken from published literature [23]. Density distribution plots represent the proportion of taxa in each category exhibiting a given nutrient response and are based on number of unique taxa in each life history strategy category. **A** The difference in ^18^O enrichment between carbon-amended soils (1000 μg-C-glucose per g dry soil) and control soils (∆^18^O EAF_C_). **B** The ∆^18^O EAF between carbon and nitrogen-amended soils (glucose + 100 μg-N [NH_4_]_2_SO_4_ per g dry soil) and control soils (∆^18^O EAF_CN_). **C** The ^13^C EAF of C-amended soils (^13^C EAF_C_). **D** The ^13^C EAF of CN-amended soils (^13^C EAF_CN_).

When comparing the difference in isotope assimilation of bacterial taxa in response to nutrients, we observed substantial overlap between the response of expected oligotrophs and expected copiotrophs and little bimodal tendency either across all phyla or within phyla (Fig. 2), aligning with hypothesis H_0_ (Fig. 1A). Accounting for shared taxonomy and differences across sites using hierarchical linear models, expected life history strategies (copiotrophic, oligotrophic, or undefined; Table 1) were a non-significant predictor of individual bacterial responses to nutrients regardless of treatment or isotopic tracer (Δ^18^O C: *F*_*2*_, _*8*_ = 0.94, *P* = 0.43; Δ^18^O CN: *F*_*2*_, _*8*_ = 1.80, *P* = 0.23; ^13^C: *F*_*2*_, _*8*_ = 1.49, *P* = 0.28; ^13^CN: *F*_*2*_, _*8*_ = 2.81, *P* = 0.12). However, we did observe ASVs with strong positive responses to C and CN addition, despite the prevailing unimodal pattern (Fig. 2B, D); and these ASVs tended to come from lineages with expectations for copiotrophic growth [23]. Bacterial ASVs with strongly positive nutrient response were constituents of the Gammaproteobacteria, Alphaproteobacteria, Actinobacteria, Firmicutes, and Bacteroidetes although they made up small proportions of each phylum (with the exception of the Firmicutes) (Fig. 2). The differential response of ASVs within some phyla suggests support for hypothesis H3 (Fig. 1C).

### Mixture models produce plausible delinations of life history strategy, but identify few consistently copiotrophic taxa

**Fig. 3:**
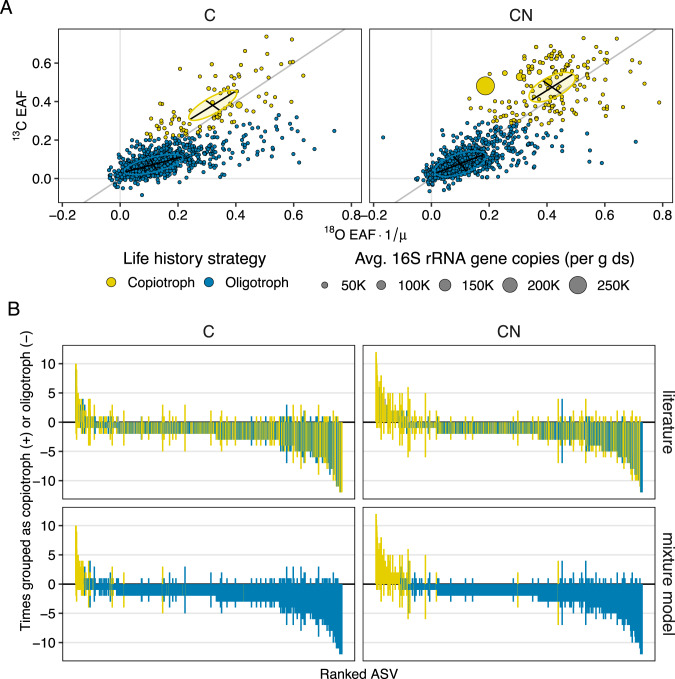
Consistency of nutrient responses across microbial life history strategies. **A** Points represent multi-isotopic excess atom fraction (EAF) of individual bacterial taxa, based on parallel seven-day ^13^C and ^18^O incubations, and averaged across all replicates in a given treatment (*n* = 12). Colors represent approximations of ecological life history strategies generated from bivariate Gaussian finite mixture models and specifying two components. Ellipsoides represent 95% confidence intervals around cluster centroids while black lines indicate principal coordinates axes of clusters. Soil treatments (C and CN) represent a carbon amendment (1000 μg glucose per g dry soil) and a carbon and nitrogen amendment (glucose + 100 μg-N [NH_4_]_2_SO_4_ per g dry soil) respectively. For direct comparison with ^13^C, EAF values of ^18^O were divided by 0.6 (µ) to account for multiple oxygen sources utilized during bacterial growth. **B** Number of times bacterial taxa across replicates were clustered into the copiotrophic category (positive values) or into the oligotrophic category (negative values). Bars are colored by life history classifications made from the most frequent classification of phyla in the literature as collected by Ho et al. [23] (top) or made from mixture model clusters in part A (bottom).

Bivariate Gaussian finite mixture modeling of joint ^18^O-^13^C growth signatures of bacterial ASVs produced clusters with similar configurations in both treatments (Fig. 3A). Slow-growing (i.e., oligotrophic) ASVs had mean enrichment values under 0.15 ($\bar x$_18O_ = 0.12, $\bar x$_13C_ = 0.07) while fast-growing (i.e., copiotrophic) ASVs had mean enrichment values greater than 0.3 ($\bar x$_18O_ = 0.32, $\bar x$_13C_ = 0.38). In both the C and CN treatments, most bacterial ASVs (>90%) were clustered into the oligotrophic growth category defined by the mixture model (as based on their treatment-averaged ^13^C and ^18^O enrichment values) (Fig. 3B).

Bacteria generally behaved consistently across replicates, but few ASVs could be identified as soley copiotrophic or oligotrophic with a high degree of statistical confidence. Nevertheless, assignment of bacterial ASVs into different life history clusters, based on groupings from mixture models (Fig. 3A), provided a clearer demarcation of behavior than expectations of life history strategy from the literature (Fig. 3B). Although, the vast majority of ASVs either clustered into some mix of copiotrophic and oligotrophic responses, or occurred too infrequently to assign a single life history strategy that was statistically significant. Per-replicate behavior significantly matched with treatment-level expectations for only 28 ASVs based on exact binomial tests (Supplemental Table 1). Of those, only three ASVs could be significantly grouped into the copiotrophic cluster (Firmicutes: *Paenibacillus* and an unclassified genus within the order Bacillales, Actinobacteria: an unclassified genus within the Micrococcaceae) while the remaining occurred in the oligotrophic cluster.

### Life history strategy at fine taxonomic levels is necessary to accurately describe taxon-specific nutrient response

**Fig. 4:**
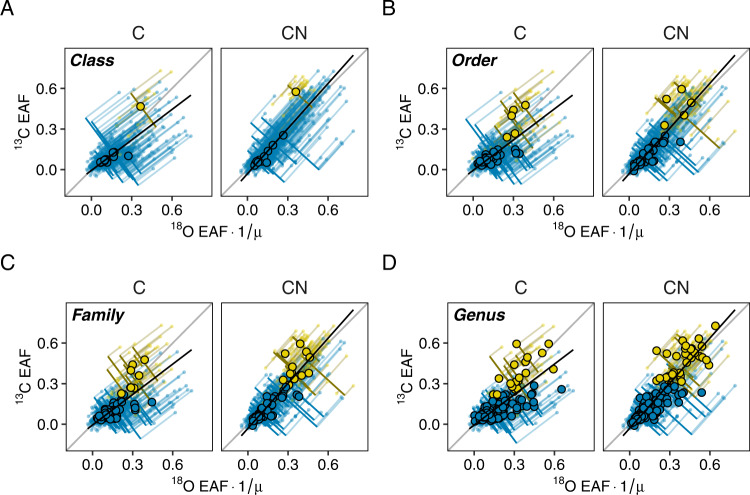
Classification of bacterial nutrient response based on averages at different taxonomic levels. Outlined points represent the isotopic enrichment (^13^C and ^18^O) of bacterial lineages at specified taxonomic levels over a 7-day incubation, based on the mean excess atom fraction (EAF) of their constituent taxa (shown by small points with lines to their representative group). Colors represent approximations of ecological life history strategies generated from bivariate Gaussian finite mixture models and specifying two components. Panels show classification of bacterial taxa based on isotopic composition of: **A** Classes, **B** Orders, **C** Families, and **D** Genera. Black lines represent the first axes generated from principal components analyses of ^18^O and ^13^C EAF values in each treatment, expressing a composite measure of bacterial growth. Lines perpendicular to the principal component represent average growth of representative lineages while smaller lines parallel to the principal component represent the differences between growth of representative lineages and individual amplicon sequence variants. Soil treatments (C and CN) represent a carbon amendment (1000 μg glucose per g dry soil) and a carbon and nitrogen amendment (glucose + 100 μg-N [NH_4_]_2_SO_4_ per g dry soil) respectively. For direct comparison with ^13^C, EAF values of ^18^O were divided by 0.6 (µ) to account for multiple oxygen sources utilized during bacterial growth.

Comparison of hierarchical linear models with life history categorizations at different taxonomic resolution indicated that finer levels were more predictive of nutrient response behavior. Assignment at the phylum and class levels, based on multi-isotopic mixture modeling clusters (Fig. 3A), produced nearly identical models that were both significantly better than site and taxonomic information alone (likelihood ratio tests; C response: *L* = 12.5, *P* < 0.001; CN response: *L* = 10.0, *P* = 0.0015), providing evidence against hypothesis H_0_ (Table 2). In both the phylum-level and class-level models, the Firmicutes and Bacilli (as a class within the Firmicutes) were the only respective lineages designated as copiotrophic, despite many ASVs showing strongly positive enrichment (Fig. 4A). At finer taxonomic resolution, the number of lineages designated as copiotrophic broadened, and models were stronger predictors of bacterial growth (Table 2) (Fig. 4). The strongest improvement was at the genus level (likelihood ratio tests; C response: order *L* = 39.7, *P* < 0.001; family *L* = 54.7, *P* < 0.001; genus *L* = 105.0, *P* < 0.001; CN response: order *L* = 39.3, *P* < 0.001; family *L* = 64.3, *P* < 0.001; genus *L* = 168.8, *P* < 0.001). This provides strong support for hypothesis H_2_ (Fig. 1C) in that life history assignments of bacterial genera may be useful in predicting nutrient response (Supplemental Figs. 3, 4) (Supplemental Data 1).

**Table 2:** Comparison of models explaining microbial nutrient response with microbial life history strategy defined at different taxonomic levels.

Assignment of life history strategy	df	ΔAIC_C-trt_	ΔAIC_CN-trt_
Genus	9	0	0
Family	9	50.3	104.5
Order	9	65.3	129.5
Class	9	92.5	158.8
Phylum	9	92.5	158.8
No life history strategy	8	103.0	166.8

Results from the hierarchical linear models on the responses of bacterial amplicon sequence variants (ASVs) to carbon (C-trt) and carbon and nitrogen (CN-trt) addition. ΔAIC represents the model fit of hierarchical models describing the relationship between joint ^18^O–^13^C enrichment (condensed via principal components analysis) and life history strategy (i.e., whether organisms were identified as oligotrophic or copiotrophic). Life history strategy of ASVs was inferred based on whether the average measured nutrient response of a taxonomic group was clustered into a high or low growth category as determined by gaussian finite mixture models. Life history strategies were made at increasingly finer levels of taxonomic organization (i.e., phylum, class, order, family, and then genus). df indicates model degrees of freedom.

## Discussion

Our results indicate that microbial life history strategies, as currently conceptualized, do not provide a strong predictive framework on the behavior and activity of most microorganisms in the soil environment. Rather, under the conditions of this experiment, microorganisms exhibited a continuous distribution from copiotrophic to oligotrophic strategies as represented from high to low growth rates, with most microorganisms showing low to intermediate growth rates. While this suggests support for our null hypothesis, H_0_, we found evidence for bimodality when considering the growth rates of a small subset of ASVs in the community which in several cases were also highly abundant. With the exception of the Firmicutes, we observed little evidence distinguishing any bacterial phylum as strongly copiotrophic or oligotrophic (as expected under hypotheses H_1_ and H_3_), indicating that assumptions about the behavior of any particular taxon cannot be made on the basis of its representative phylum. Fierer et al. note as much in their seminal work [6].

Copiotrophic and oligotrophic modes are roughly analogous to nutrient acquisition or stress tolerance strategies (respectively) within the yield-acquisition-stress tolerance framework [10]. The assumptions of the YAS framework indicate that more complex substrates may be better suited to differentiating nutrient acquisitive microorganisms (copiotrophs) from others. While the labile nutrients supplied in this study, glucose and ammonium sulfate, were intended to serve as a proxy for plant root exudates in a priming experiment [45], they did not truly represent the diversity and complexity of native substrates that would be expected in a copiotrophic soil environment. Thus, the ability of this study to address broad hypotheses about life history strategies across the bacterial tree of life may be limited.

We saw significant improvement in hierarchical linear models of nutrient response when life history strategy was estimated at finer levels of taxonomic resolution (e.g., family and especially genus) which indicates strong support for hypothesis H_2_. Therefore, while we refute the continued use of categorical assumptions of oligotrophic or copiotrophic life histories for bacterial phyla, our in situ findings suggest that such representations could be useful if made at the genus level (although perhaps only in the context of artificial resource amendment) – in agreement with previously reported conclusions [23].

Despite the best performance of the genus-level models to estimate nutrient responses, we had difficulty confidently characterizing the growth of individual ASVs; few could be consistently labeled as copiotrophs because many grew both quickly and slowly across the replicates in our experiment. Such indeterminancy is partially due to the low sample size of our experiment, but also likely stems from the inherent stochasticity of the soil environment. This context-dependency of bacterial responses (either by nutrient complexity and character or by local ecosystem characteristics) is another argument against categorical application of life history strategy at a broad taxonomic level. For example, it will be difficult to predict the growth of a “copiotroph” if its behavior depends on a complex arrangement of soil characteristics, nutrient availability, and local biotic interactions rather than more relatively static traits such as 16S rRNA gene copy number or genome size.

The soils used in the current experiment were subject to considerable disturbance including physical disruption, dry-down, and sudden wet-up, inducing a strong pattern of microbial turnover, activity, and respiration from new organic matter made available to the soil community [25, 45–47]. As such, we employed ^13^C-glucose additions to track its utilization specifically. We found high shared variation between ^18^O and ^13^C EAF values, suggesting that most microorganisms utilized the added glucose. Further, ^18^O and ^13^C EAF values covaried more strongly in the CN treatment, suggesting that N limitation may have limited glucose uptake in the C treatment. Thus, microorganisms with high ^18^O signatures but low ^13^C signatures may be those with high N demand who prioritized the decomposition of native soil organic matter to meet their needs. However, the presence of microorganisms with high ^18^O but low ^13^C signatures in the CN treatment suggests that N limitation alone may not explain why some microorganisms did not utilize the added glucose. Besides mixing, successional dynamics across the incubation may also explain differences in isotopic signatures of bacteria. Thus, we took this into account in our mixture model specifications such that these microorganisms (high ^18^O but low ^13^C EAF values) were clustered as oligotrophic, based on the possibility that they grew after the depletion of added glucose.

Our results do suggest that – in our aerobic mineral soils at least – the potential for quick growth in response to labile nutrients exists within a small portion of the bacterial community and that this potential seems to be phylum-specific. Among these phyla, however, it is more accurate to understand nutrient response as a continuum rather than a dichotomous classification. If classification is necessary for statistical or narrative purposes, we recommend to restrict life history designations to the family or genus level. These findings (produced by qSIP) were measured by within the context of microbial community interactions which is an important line of inference to better understand microbial trait adaptations. In keeping with other ecological frameworks (e.g., C-S-R and Y-A-S [10, 48]), stress treatments are a priority for future studies in order to understand the diversity of stress tolerance strategies and their effect on growth. The utilization of both simple and complex nutrient sources across the community (as well as from both plant and microbial origin) will also be a key point of inquiry, and designs that explore this difference will refine our thinking of microbial ecology in the soil realm (e.g., Dang et al. [49]). Lastly, the relatively short timescales inherent to nutrient pulse-type experiments mean that such incubations must be placed into longer-term studies strategically. For example, repeated or long-term amendments with both qSIP and complimentary 16S rRNA gene surveys can show how well short-term growth rates relate to stable community adaptation. Plant ecologists have embraced trait-based approaches, such as the application of leaf economic spectrum as an important predictor of global carbon flux within a larger framework of interrelated trait dimensions and trade-offs [50]. Correspondingly, future trait explorations in microbial ecology should also be paired with measures of nutrient and energy fluxes to link community composition with ecosystem dynamics.

## Supplementary information

Supplemental Information

Bacterial Life History Strategy and Taxonomy

Experimental data

## Data Availability

DNA sequence data and sample metadata have been deposited in the NCBI Sequence Read Archive under the project ID PRJNA521534. Experimental data have been included as a supplement to this publication.
