# Supplementary material for: Life history strategies among soil bacteria—dichotomy for few, continuum for many
Source: ISME J. 2023 Feb 2;17(4):611–9. doi: 10.1038/s41396-022-01354-0 (PMC10030646; doi:10.1038/s41396-022-01354-0)
Supplement: Supplementary file 1 — Supplemental Information [file 41396_2022_1354_MOESM1_ESM.pdf]

Supplemental information to: Life history strategies among soil bacteria – dichotomy for few, continuum for many

Bram W. G. Stone[1,2], Paul Dijkstra[2,3], Brianna K. Finley[4], Raina Fitzpatrick[2], Megan M. Foley[2,3], Michaela Hayer[2], Kirsten S. Hofmockel[1,5], Benjamin J. Koch[2,3], Junhui Li[2,6], Xiao Jun A. Liu[7], Ayla Martinez[2], Rebecca L. Mau[2], J ane Marks[2,3], Victoria Monsaint-Queeney[2], Ember M. Morrissey[8], Jeffrey Propster[2], Jennifer Pett-Ridge[9,10], Alicia M. Purcell[2,3], Egbert Schwartz[2,3], Bruce A. Hungate[2,3]

1. Earth and Biological Sciences Directorate, Pacific Northwest National Lab, Richland WA, USA
2. Center for Ecosystem Science and Society, Northern Arizona University, Flagstaff, AZ, USA
3. Department of Biological Sciences, Northern Arizona University, Flagstaff, AZ, USA
4. Department of Ecology and Evolutionary Biology, University of California, Irvine, CA, USA
5. Department of Agronomy, Iowa State University, Ames, IA, USA
6. APC Microbiome Ireland and School of Microbiology, University College Cork, Ireland
7. Institute for Environmental Genomics, Department of Microbiology and Plant Biology, University of Oklahoma, Norman, OK, USA.
8. Division of Plant and Soil Sciences, West Virginia University, Morgantown, WV, USA
9. Physical and Life Sciences Directorate, Lawrence Livermore National Lab, Livermore, CA, USA
10. Life and Environmental Sciences Department, University of California Merced, Merced, CA, USA

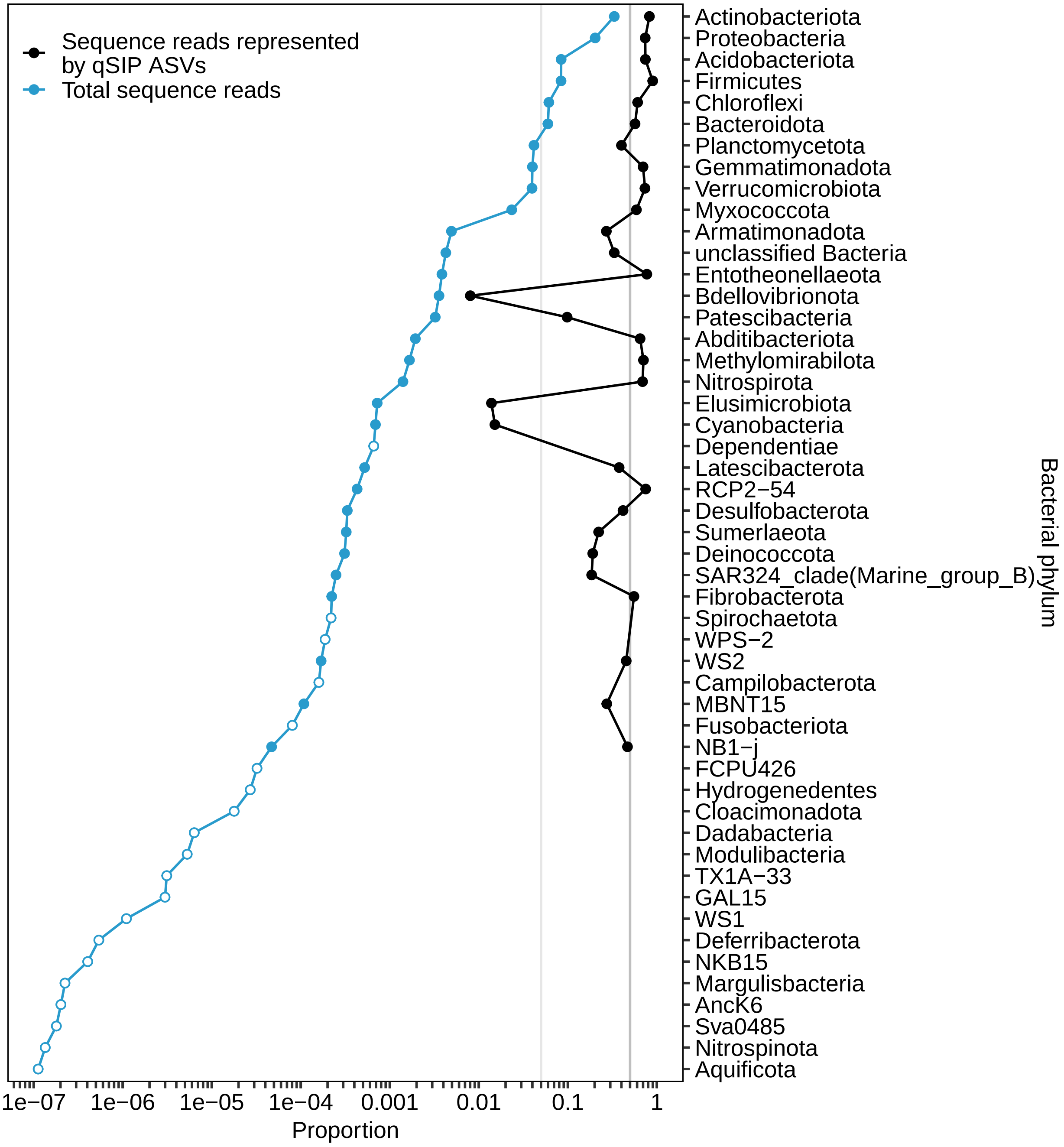

Supplemental Figure 1. Representativeness of quantitative stable isotope probing (qSIP) dataset. Points represent the proportional abundance of 16S rRNA gene sequence reads classified to a particular bacterial phylum. Blue points represent the typical quantification of proportional abundance across the sequencing data. Black points represent the proportion of sequence reads from each bacterial phylum that are retained in the qSIP data set after filtering to remove infrequent taxa. Open points represent bacterial phyla which were present in the full sequencing data but not in the qSIP-filtered subset.

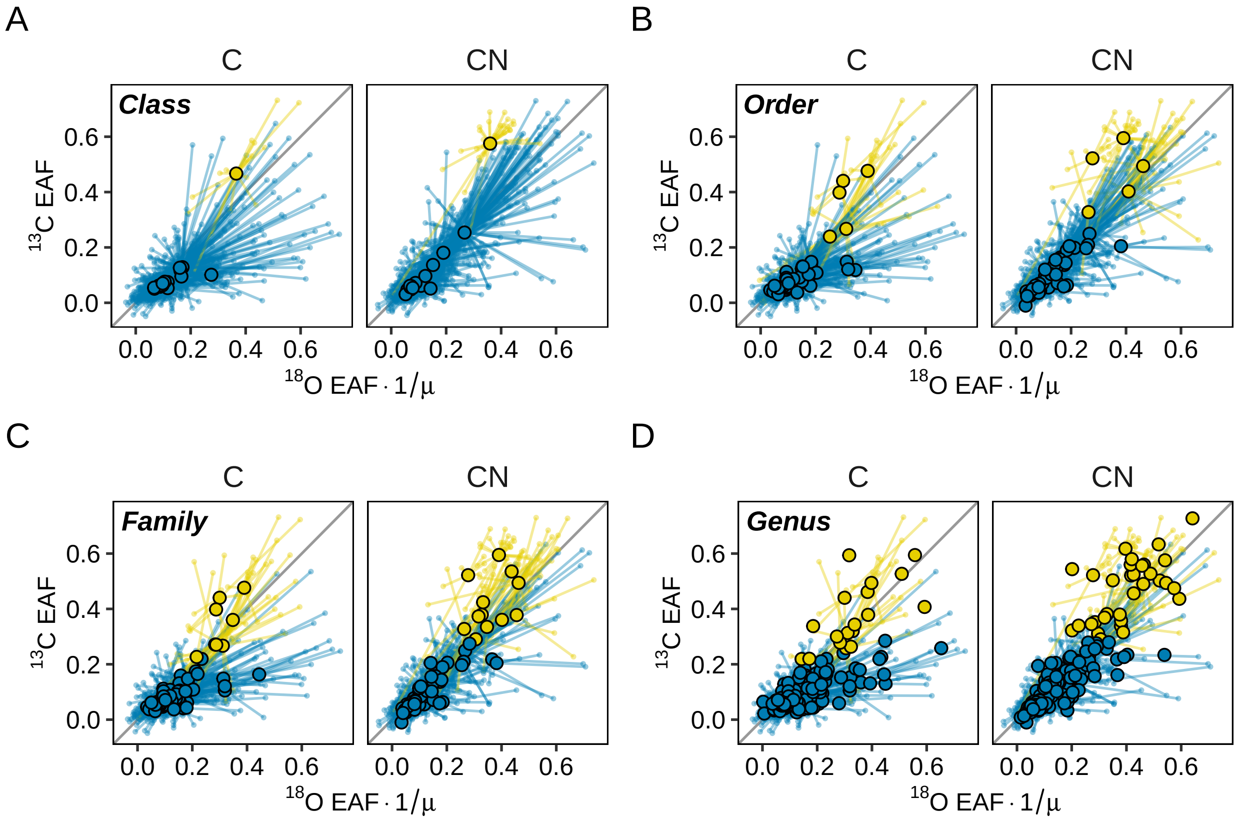

Supplemental Figure 2. Classification of bacterial nutrient response based on averages at different taxonomic levels. Outlined points represent multi-isotopic excess atom fraction (EAF) of bacterial lineages, measured from parallel seven-day ^{13}C and ^{18}O incubations, based on the enrichment of their constituent taxa (small points with lines to representative lineage). Colors represent approximations of ecological life history strategies generated from bivariate Gaussian finite mixture models and specifying two components. Panels show classification of bacterial taxa based on isotopic composition of: **A)** Classes, **B)**, Orders, **C)** Families, and **D)** Genera. Soil treatments (C and CN) represent a carbon amendment (1000 μg glucose per g dry soil) and a carbon and nitrogen amendment (glucose + 100 μg-N $[NH_4]_2SO_4$ per g dry soil) respectively. For direct comparison with ^{13}C, EAF values of ^{18}O were divided by 0.6 (μ) to account for multiple oxygen sources utilized during bacterial growth.

Taxon ID	Phylum	Class	Order	Family	Genus	Strategy from mixture models	trt	*P* value (FDR-adjusted)
ASV1	Actinobacteriota	Thermoleophilia	Solirubrobacterales	Solirubrobacteraceae	unclassified Solirubrobacteraceae	Oligotroph	C	0.031
ASV2	Actinobacteriota	Thermoleophilia	Solirubrobacterales	Solirubrobacteraceae	Solirubrobacter	Oligotroph	C	0.032
ASV3	Bacteroidota	Bacteroidia	Chitinophagales	Chitinophagaceae	unclassified Chitinophagaceae	Oligotroph	C	0.031
ASV4	Acidobacteriota	Blastocatellia	Blastocatellales	Blastocatellaceae	unclassified Blastocatellaceae	Oligotroph	C	0.032
ASV5	Actinobacteriota	Thermoleophilia	Solirubrobacterales	67-14	67-14	Oligotroph	C	0.031
ASV6	Methylomirabilota	Methylomirabilia	Rokubacteriales	Rokubacteriales	Rokubacteriales	Oligotroph	C	0.032
ASV7	Alphaproteobacteria	Alphaproteobacteria	Sphingomonadales	Sphingomonadaceae	unclassified Sphingomonadaceae	Oligotroph	C	0.031
ASV8	Actinobacteriota	Thermoleophilia	Solirubrobacterales	67-14	67-14	Oligotroph	C	0.031
ASV9	Verrucomrobiota	Verrucomrobiae	Chthoniobacterales	Chthoniobacteraceae	Candidatus Udaeobacter	Oligotroph	C	0.032
ASV10	Acidobacteriota	Blastocatellia	Pyrinomonadales	Pyrinomonadaceae	RB41	Oligotroph	C	0.031
ASV11	Actinobacteriota	Actinobacteria	Frankiales	Geodermatophilaceae	Blastococcus	Oligotroph	C	0.031
ASV12	Actinobacteriota	Thermoleophilia	Solirubrobacterales	Solirubrobacteraceae	Solirubrobacter	Oligotroph	C	0.032
ASV13	Actinobacteriota	Thermoleophilia	Solirubrobacterales	67-14	67-14	Oligotroph	C	0.031
ASV14	Chloroflexi	Chloroflexia	Thermomrobiales	JG30-KF-CM45	JG30-KF-CM45	Oligotroph	C	0.032
ASV15	Acidobacteriota	Blastocatellia	Pyrinomonadales	Pyrinomonadaceae	RB41	Oligotroph	C	0.032
ASV16	Acidobacteriota	Blastocatellia	Pyrinomonadales	Pyrinomonadaceae	RB41	Oligotroph	C	0.031
ASV17	Verrucomrobiota	Verrucomrobiae	Chthoniobacterales	Chthoniobacteraceae	Candidatus Udaeobacter	Oligotroph	C	0.032
ASV18	Actinobacteriota	Actinobacteria	Frankiales	Geodermatophilaceae	Geodermatophilus	Oligotroph	C	0.031
ASV19	Gemmatimonadota	Gemmatimonadetes	Gemmatimonadales	Gemmatimonadaceae	unclassified Gemmatimonadaceae	Oligotroph	C	0.031
ASV20	Gemmatimonadota	Gemmatimonadetes	Gemmatimonadales	Gemmatimonadaceae	unclassified Gemmatimonadaceae	Oligotroph	C	0.032
ASV21	Actinobacteriota	Actinobacteria	Frankiales	Geodermatophilaceae	Geodermatophilus	Oligotroph	C	0.031
ASV22	Actinobacteriota	Actinobacteria	Mromonosporales	Mromonosporaceae	unclassified Mromonosporaceae	Oligotroph	C	0.032
ASV23	Actinobacteriota	Thermoleophilia	Solirubrobacterales	Solirubrobacteraceae	Solirubrobacter	Oligotroph	C	0.032
ASV5	Actinobacteriota	Thermoleophilia	Solirubrobacterales	67-14	67-14	Oligotroph	CN	0.033
ASV6	Methylomirabilota	Methylomirabilia	Rokubacteriales	Rokubacteriales	Rokubacteriales	Oligotroph	CN	0.047
ASV24	Firmutes	Bacilli	Bacillales	unclassified Bacillales	unclassified Bacillales	Copiotroph	CN	0.033
ASV7	Alphaproteobacteria	Alphaproteobacteria	Sphingomonadales	Sphingomonadaceae	unclassified Sphingomonadaceae	Oligotroph	CN	0.033

ASV25	Actinobacteriota	Actinobacteria	Mrococcales	Mrococcaceae	unclassified Mrococcaceae	Copiotroph	CN	0.033
ASV9	Verrucomrobiota	Verrucomrobiae	Chthoniobacterales	Chthoniobacteraceae	Candidatus Udaeobacter	Oligotroph	CN	0.033
ASV10	Acidobacteriota	Blastocatellia	Pyrinomonadales	Pyrinomonadaceae	RB41	Oligotroph	CN	0.033
ASV13	Actinobacteriota	Thermoleophilia	Solirubrobacterales	67-14	67-14	Oligotroph	CN	0.047
ASV26	Alphaproteobacteria	Alphaproteobacteria	Rhizobiales	Xanthobacteraceae	Bradyrhizobium	Oligotroph	CN	0.033
ASV16	Acidobacteriota	Blastocatellia	Pyrinomonadales	Pyrinomonadaceae	RB41	Oligotroph	CN	0.033
ASV17	Verrucomrobiota	Verrucomrobiae	Chthoniobacterales	Chthoniobacteraceae	Candidatus Udaeobacter	Oligotroph	CN	0.033
ASV27	Alphaproteobacteria	Alphaproteobacteria	Sphingomonadales	Sphingomonadaceae	Sphingomonas	Oligotroph	CN	0.033
ASV19	Gemmatimonadota	Gemmatimonadetes	Gemmatimonadales	Gemmatimonadaceae	unclassified Gemmatimonadaceae	Oligotroph	CN	0.047
ASV28	Firmutes	Bacilli	Paenibacillales	Paenibacillaceae	Paenibacillus	Copiotroph	CN	0.047

Supplemental Table 1. Taxonomic identification of bacterial taxa with consistent life history behavior. Soils were amended with 1000 µg-C glucose per g dry soil (C treatment) or with glucose + 100 µg-N $[NH_4]_2SO_4$ per g dry soil (CN treatment). Taxa included represent 28 distinct bacterial amplicon sequence variants (ASVs) whose growth across 12 replicates clustered into the same group (slow-growth = oligotroph, fast-growth = copiotroph) as expected based on treatment-level average growth and Gaussian finite mixture model clustering (column: "Strategy from mixture models"). Significant agreement between per-replicate clustering and treatment-level clustering was determined by binomial exact tests ($\alpha = 0.05$) and *P* values were adjusted using the false-discovery rate method to limit type I error. Soil treatments are designated by the "trt" column.

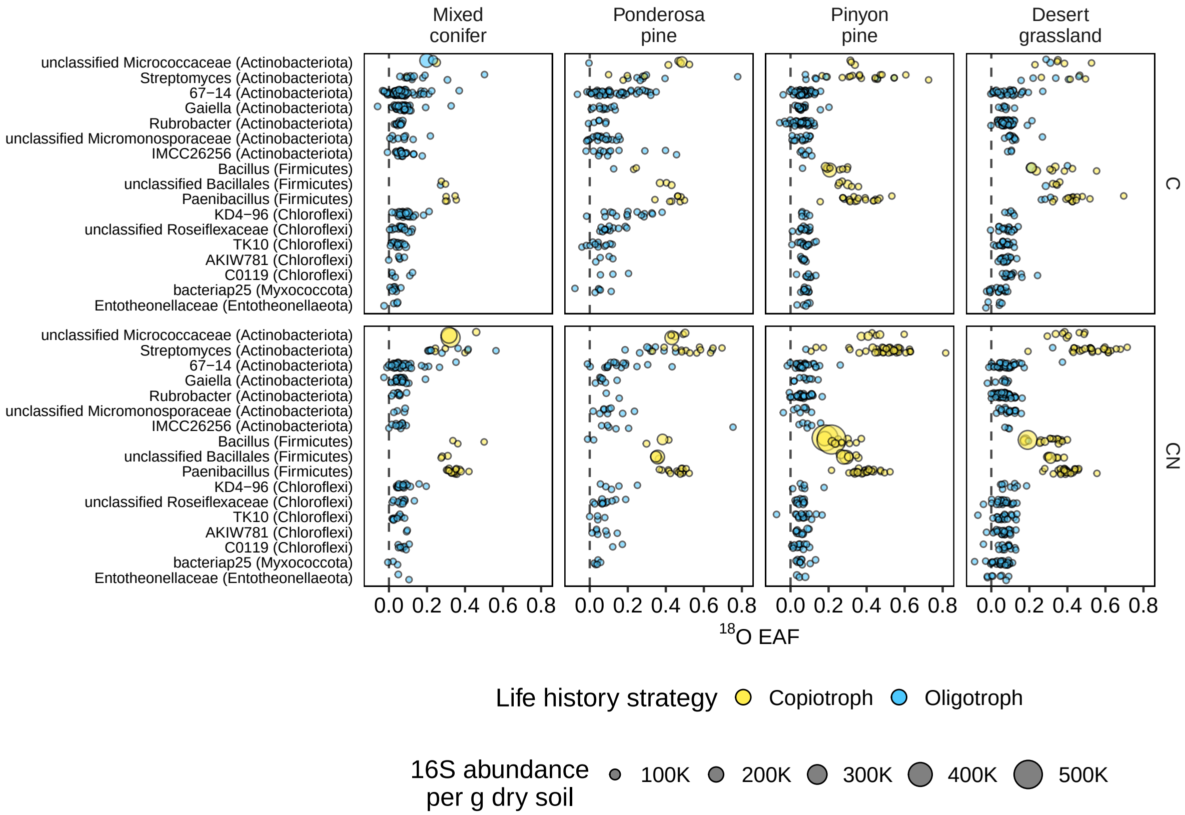

Supplemental Figure 3. Bacterial response to nutrient addition across genera. Each point represents the ^{18}O excess atom fraction (^{18}O EAF) for an individual bacterial taxon averaged across three soil replicates sampled from one of four ecosystems (top). Points are colored by the categorical assignment of life history strategy applied at the phylum level based on Gaussian finite mixture modeling of treatment-level average EAF values. Soil treatments (C and CN, right) represent a carbon amendment (1000 μg glucose per g dry soil) and a carbon and nitrogen amendment (glucose + 100 μg-N $[NH_4]_2SO_4$ per g dry soil) respectively. Bacterial genera are named with representative phyla in parentheses. Genera were selected as those that make up more than 75% of 16S rRNA gene sequence reads.

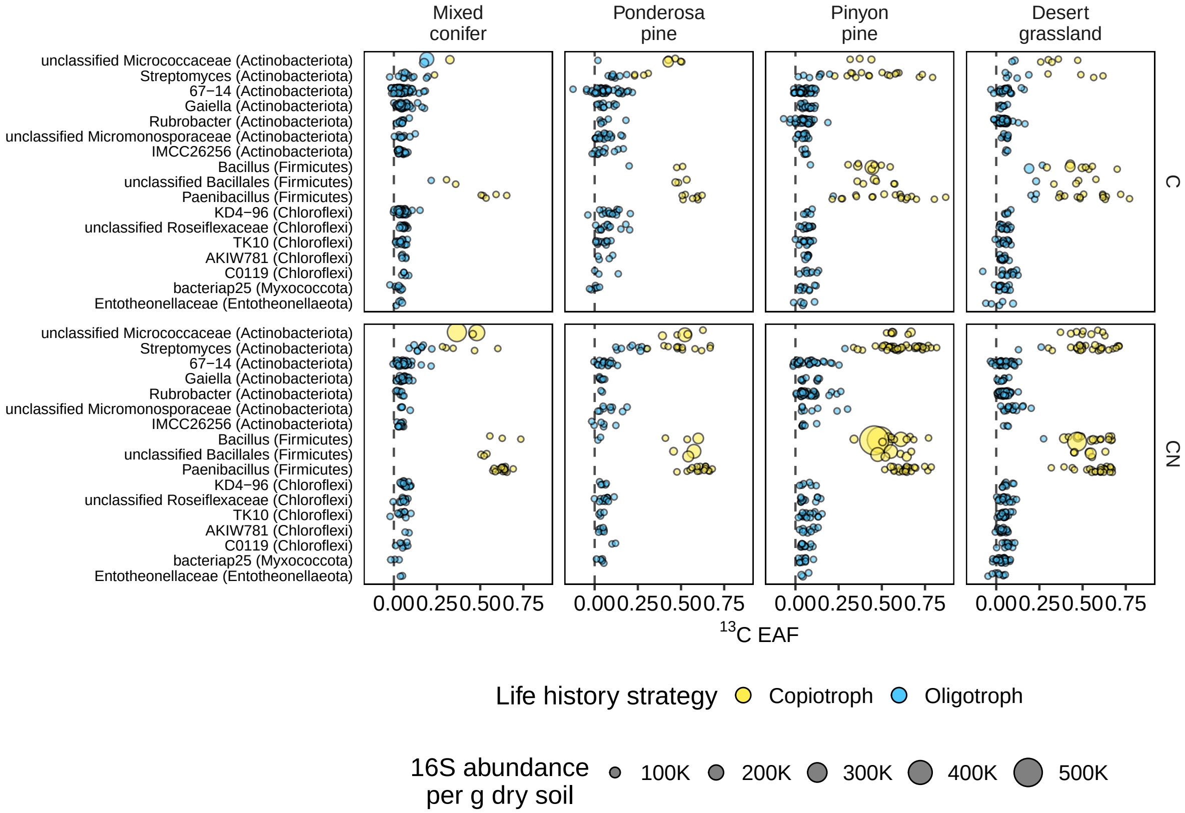

Supplemental Figure 4. Bacterial response to nutrient addition across genera. Each point represents the ^{13}C excess atom fraction (^{13}C EAF) for an individual bacterial taxon averaged across three soil replicates sampled from one of four ecosystems (top). Points are colored by the categorical assignment of life history strategy applied at the phylum level based on Gaussian finite mixture modeling of treatment-level average EAF values. Soil treatments (C and CN, right) represent a carbon amendment (1000 μg-^{13}C glucose per g dry soil) and a carbon and nitrogen amendment (^{13}C-glucose + 100 μg-N $[NH_4]_2SO_4$ per g dry soil) respectively. Bacterial genera are named with representative phyla in parentheses. Genera were selected as those that make up more than 75% of 16S rRNA gene sequence reads.
